# Hamstring muscle activation strategies during eccentric contractions are related to the distribution of muscle damage

**DOI:** 10.1111/sms.14191

**Published:** 2022-06-06

**Authors:** Valentin Goreau, Robin Pigne, Nathan Bernier, Antoine Nordez, François Hug, Lilian Lacourpaille

**Affiliations:** ^1^ Movement–Interactions–Performance, MIP, UR 4334 Nantes Université Nantes France; ^2^ CIAMS Université d'Orléans Orléans France; ^3^ CIAMS Université Paris‐Saclay Orsay France; ^4^ Institut Universitaire de France (IUF) Paris France; ^5^ LAMHESS Université Côte d'Azur Nice France

**Keywords:** DOMS, electromyography, muscle coordination, shear modulus, shear wave elastography

## Abstract

Large inter‐individual variability of activation strategies is observed during hamstring strengthening exercises but their consequences remain unexplored. The objective of this study was to determine whether individual activation strategies are related to the distribution of damage across the hamstring muscle heads semimembranosus (SM), semitendinosus (ST), and biceps femoris (BF) after eccentric contractions. 24 participants performed 5 sets of 15 maximal eccentric contractions of knee flexors on a dynamometer, while activation of each muscle head was assessed using surface electromyography. Knee flexion maximal isometric strength was assessed before exercise and 48 h afterward. Shear modulus was measured using shear wave elastography before exercise and 30 min afterward to quantify the distribution of damage across the hamstring muscle heads. At 48 h, maximal knee flexion torque had decreased by 15.9% ± 16.9% (*p* < 0.001). Although no differences between activation ratios of each muscle were found during the eccentric exercise (all *p* > 0.364), we reported a heterogeneous distribution of damage, with a larger change in shear modulus of ST/Hams than SM/Hams (+70.8%, *p* < 0.001) or BF/Hams (+50.3%, *p* < 0.001). A large correlation was found between the distribution of activation and the distribution of damage for ST/Hams (*r* = 0.69; *p* < 001). This study provides evidence that the distribution of activation during maximal eccentric contractions has mechanical consequences for synergist muscles. Further studies are needed to understand whether individual activation strategies influence the distribution of structural adaptations after a training program.

## 1 INTRODUCTION

There is a consensus in the literature to recommend the use of eccentric exercises in hamstring injury prevention programs.^1^, ^2^ During such exercises, high inter‐individual variability of activation strategies has recently been observed.^3^ For instance, 30% of the participants exhibited a higher activation of the biceps femoris during the stiff‐leg Deadlift exercise, while 35% showed higher activation of the semimembranosus.^3^ These results are in accordance with studies performed during locomotor tasks, demonstrating that each individual has a unique muscle activation signature.^4^, ^5^ However, the mechanical effects of such muscle activation signatures remain poorly understood. As muscle activation influences muscle stress (i.e., force per cross‐sectional area^6^), one consequence of differing activation signatures would be a heterogeneous distribution of muscle damage between individuals after maximal eccentric exercise. Therefore, it is likely that some activation signatures favor the damage of a specific muscle within a muscle group.

Some studies have reported the distribution of damage among hamstring muscles induced by eccentric exercise.^7^, ^8^, ^9^, ^10^ All these studies observed that the semitendinosus (ST) muscle suffers a greater amount of muscle damage than the biceps femoris (BF) and semimembranosus (SM). However, the origin of this inter‐muscular heterogeneity and the putative interindividual variability of this distribution of muscle damage remains unknown. Addressing these questions is important because muscle damage induced by resistance training could provide us with information about the amount of stress applied to each individual muscle, which is one of the main triggers of structural adaptations.^11^

A series of studies have shown that the muscle shear modulus (index of muscle stiffness) increases 30 min after eccentric exercise^12^, ^13^, ^14^ and that this increase is correlated with the magnitude of strength deficit measured at 48 h after this exercise.^13^ It is important to note that the shear modulus values return to baseline values at 48 h after exercise or remain slightly elevated for some muscles in stretched positions.^12^, ^14^ Overall, considering that the strength deficit 48 h after the damage is a good indicator of the amount of damage,^15^ changes in shear modulus measured at 30 min after exercise has been proposed as a non‐invasive tool to detect the distribution of damage among synergist muscles.^13^

The objective of this study was to determine whether individual activation strategies influence the distribution of damage across the heads of the SM, ST, and BF hamstring muscles after eccentric contractions. We hypothesize that, for a given muscle, the distribution of activation is related to the distribution of muscle damage among hamstring heads. Although the increase in shear modulus is an indirect outcome of the amount of damage in individual muscle,^12^, ^13^, ^16^, ^17^ we used the term distribution of muscle damage instead of the distribution of changes in shear modulus throughout the manuscript for sake of clarity.

## 2 MATERIALS AND METHODS

### 2.1 Participants

A total of 24 healthy participants, 14 men (26.4 ± 1.9 years; 79.1 ± 12.8 kg; 184.9 ± 5.6 cm) and 10 women (25.5 ± 2.3 years; 63.2 ± 9.8 kg; 168.3 ± 5.7 cm), were recruited on a voluntary basis after providing written informed consent. They were not involved in a physical activity involving intense hamstring eccentric contractions (e.g., sprinting, resistance training, etc.). None of the participants had a history of lower limb injury that had limited function or required them to seek intervention from a health care professional. Participants did not consume any medication and/or nutritional supplementation that may alter recovery after exercise. The experimental procedures were approved by the local Ethics Committee (CPP IDF I, n°2018‐A02675‐50), and all procedures adhered to the Declaration of Helsinki.

### 2.2 Protocol

**FIGURE 1:**
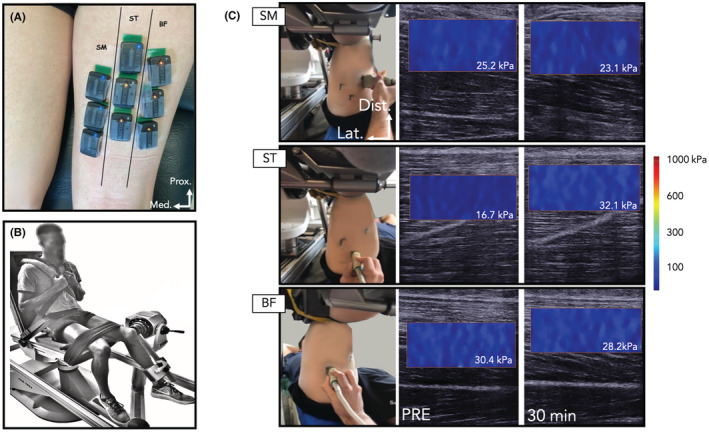
Typical examples of electrode positioning (A), experimental setup (B), and shear modulus maps (C). Shear modulus of the hamstring heads (semimembranosus, SM; semitendinosus, ST; biceps femoris, (BF)) was measured before the eccentric exercise (PRE) and 30 min afterward (30 min)

The experiment was carried out in two sessions spaced 48 h apart. The first session lasted ~3 h and the second ~15 min. The second session was only composed of maximal isometric voluntary knee flexion contractions. The eccentric protocol was performed in the first session, on the participants' dominant leg, that is, the leg used to kick a ball. It consisted of 5 sets of 15 repetitions of isokinetic eccentric knee flexion in a seated position (hip = 70°; 0° = lying supine) (Figure 1; panel (B)). The motion ranged from 90° to 10° knee flexion (0° = knee fully extended) at a speed of 30°s^−1^. The rest time between each set was 2 min to minimize the effects of fatigue. The participants were instructed to exert as much effort as they could in the movement. During the contractions, the myoelectrical activity of hamstring muscles was measured using EMG, and torque was recorded.

Before the exercise and 48 h afterward, maximal isometric knee flexion torque was measured as it provides a good estimation of the amount of muscle damage.^18^ This measurement was performed with 70° of hip and 45° of knee flexion. Two maximal voluntary knee flexions (MVC) were performed. Then, if more than 10% of the variation was found between these first two flexions, a third was performed. For EMG normalization purposes, two additional maximal voluntary knee flexion contractions were also performed at 10° and 80° of knee flexion.^19^ During MVC, participants were asked to get to maximal strength as fast as they could and to maintain it for 3–4 s. They were strongly encouraged by the experimenter, and their myoelectrical activity was measured using EMG. The maximal voluntary contractions were performed in random order with 60 s rest in between. The greatest torque and EMG amplitude values were kept for further analysis. Participants were asked to avoid any intensive physical activity between the two sessions.

Before the eccentric protocol was performed and 30 min afterward, the shear modulus was measured using shear wave elastography on SM, ST, and BF long head. This is because strong correlations have been found in 53 participants between the strength loss at 48 h after exercise and the increase in shear modulus measured at 30 min after exercise.^13^ It is important to note that an increase in shear modulus may be observed immediately after exercise but the measurement at 30 min ensures that all the participants reach the plateau of increase in shear modulus (unpublished observations; *n* = 6). The participants were lying supine with 70° hip flexion and 45° knee flexion that placed the hamstring on the favorable limb of the joint angle‐torque relationship.^20^ Note that shear modulus was also measured at 30° of knee flexion for another purpose. Two measurements were performed for each muscle, as this methodology has shown good reliability.^21^

### 2.3 Data collection

Maximal voluntary contraction and eccentric exercise measurements were performed on an isokinetic ergometer (Con‐Trex MJ, CMV). This methodology has shown excellent inter‐day reliability when performed by the same examiner on healthy participants.^22^ All mechanical signals were provided by the dynamometer.

Shear modulus was measured for the ST, SM, and BF long head using an ultrasound scanner (Aixplorer version 12.4, Supersonic Imagine) coupled with a linear transducer array (4–15 MHz, SuperLinear 15–4; Vermon) used in shear wave elastography mode (musculoskeletal preset) as previously described in Lacourpaille et al^12^ The reliability of this technique has been previously evaluated^23^ (more details are given below; section Statistical analysis). This technique provides a two‐dimensional map of the shear modulus of a localized area in real‐time at 1 Hz. Hamstring locations known to provide the most reliable measurement were used for these shear modulus measurements.^23^ The ultrasound transducer was positioned within the plane of the fascicles for each muscle and perpendicular to the skin. This location was marked on the skin using a waterproof marker so that the transducer location remained constant between recordings (Figure 1; panel C). For each recording, muscle shear modulus was measured for 10 s and averaged over this period. The participant was instructed to remain as relaxed as possible.

The myoelectrical activity was measured using surface EMG electrodes placed over the SM, ST, and BF muscles. First, the skin was shaved and cleaned with alcohol and wireless surface electrodes (Trigno Flex, Delsys) were attached to the skin with double‐sided tape (inter‐sensor distance = 1 cm). Electrode location was checked with B‐mode ultrasound to ensure that they were positioned away from the borders of the neighboring muscles and aligned with the direction of the fascicles. To minimize crosstalk, especially between SM and ST, EMG electrodes were placed at the midline of the most prominent superficial muscle belly of each hamstring head. According to the morphology of the hamstring,^24^ the electrode locations for BF and SM were slightly more distal than that for ST (Figure 1; panel A). Regional variation in EMG amplitude has been observed when using multichannel surface EMG.^25^, ^26^ Therefore, to obtain a representative EMG measurement, we placed three electrodes over each individual muscle at proximal, middle, and distal locations, with 1–2 cm between them. This methodology has shown *excellent* inter‐day reliability during Nordic hamstring and stiff‐leg Deadlift exercises.^3^ The EMG signals were bandpass filtered (10–850 Hz, third‐order Butterworth filter) and digitized at a sampling rate of 2000 Hz (Trigno; Delsys).

### 2.4 Data analysis

The elastography data processing was performed on MATLAB software (The Mathworks, Nathicks, USA) with the ElastoGUI open software (https://bio.tools/elastogui). The region of interest was defined manually. Care was taken to exclude artifacts (saturation or void areas). Over the 144 videos recorded (24 participants, 3 muscles, and 2 trials) a total of three videos were excluded because of poor B‐mode image quality and/or elasticity map quality. The calculation of the distribution of muscle damage from changes in shear modulus is described in the section Statistical analysis.

The raw EMG signals were first bandpass filtered (20–450 Hz) with a second‐order Butterworth filter, then a 50 Hz notch filter was applied. For MVCs, the root mean square (RMS) of the EMG signal was calculated over a window of 250 ms with an overlap of 99%. For each electrode, the highest value among the three tested knee angles was considered to be the maximum RMS EMG value.^3^ For each muscle, the RMS EMG values of the three electrodes were averaged over the full range of motion of the eccentric phase during the 15 repetitions of the first set. This value was then normalized to the maximum RMS EMG to obtain the mean percentage activation per muscle (% RMS MAX EMG). The distribution of muscle activation among hamstring heads was calculated as the contribution of each muscle to the sum of the three hamstring heads (SM/Hams, ST/Hams, and BF/Hams).^27^, ^28^ Similarly, the distribution of muscle damage was calculated as the contribution of changes in shear modulus for each muscle over the sum of the value from the three hamstring heads. Indeed, it is also important to calculate the ratio of changes in shear modulus to compare the distribution of muscle damage between individuals. This is because, for a given muscle, a similar absolute increase in shear modulus between two individuals does not necessarily represent a similar distribution of muscle damage. Let us consider two participants (A and B) with an increase of about 30% in the shear modulus of the SM after the eccentric protocol. Participant A exhibits an increase in shear modulus of about 10% for both ST and BF, while participant B exhibits an increase in shear modulus of about 30% for both ST and BF. Therefore, the distribution of muscle damage is about 60%, 20%, and 20%, for SM/Hams, ST/Hams, and BF/Hams, respectively, for participant A, while it is equally distributed among hamstring heads (33%) for participant B. This ratio of changes in shear modulus was considered as the distribution of muscle damage and, thus, was only calculated for participants with muscle damage. In the participants without muscle damage, the distribution of changes in shear modulus would be random, due to errors in measurements. Therefore, we used the coefficient of variation of the shear modulus obtained in a previous reliability study (12.6%),^23^ as a threshold to detect the substantial changes in muscle shear modulus. Six participants out of the 24 participants did not reach the threshold after the eccentric exercise protocol. Note that these participants showed a mean strength loss of about 2.0% ± 3.3% of MVC at 48 h after exercise, which confirms the absence of muscle damage.^18^ Therefore, these individuals were not included in the analysis.

### 2.5 Statistical analysis

The statistical software R Version 4.1.1 (R Foundation, https://www.r‐project.org) was used for data analysis. Data are presented as the average ± standard deviation. To assess the magnitude of damage, we compared maximal knee flexion strength between before and 48 h after the eccentric exercise using a Student's *t*‐test. Due to a technical issue with the dynamometer at 48 h after the exercise for three participants, this analysis was carried out on 21 participants. To confirm the presence of muscle damage on each hamstring head, we assessed the changes in shear modulus using a two‐way repeated‐measures analysis of variance [within subject factor: time (PRE, and 30 min) and muscle (ST, SM, and BF)]. The shear modulus ratios (SM/Hams, ST/Hams, and BF/Hams) were compared using three separate Student's paired *t*‐test to determine whether the distribution of muscle damage varied between hamstring heads (*n* = 18). Three separate Student's paired *t*‐test were also used to determine whether the distribution of activation differs between the three hamstring heads during the eccentric exercise hamstring (SM/Hams, ST/Hams, and BF/Hams). When appropriate, post‐hoc analyses were performed using Bonferroni tests.

Finally, a Pearson correlation coefficient was used to determine whether the distribution of activation was correlated to the distribution of the changes in shear modulus for each muscle ratio (SM/Hams, ST/Hams, and BF/Hams) (*n* = 18). Also, a Pearson correlation coefficient was used to determine whether the distribution of activation among hamstring heads was correlated with the magnitude of the strength loss at 48 h after exercise (*n* = 21). Coefficients of correlation were considered as *negligible*, *small*, *moderate*, or *large* at <0.10, 0.11–0.30, 0.31–0.50, and >0.50, respectively.^29^

## 3 RESULTS

The maximal knee flexion torque was 126.3 ± 33.3 Nm before and 106.5 ± 35.6 Nm 48 h post‐exercise (difference 19.9 Nm, BCa 95% CI: 10.8–30.2 Nm, *p* < 0.001). This represents a strength loss of about −15.9% ± 16.9% (range: +11.0% to −45.9%).

**FIGURE 2:**
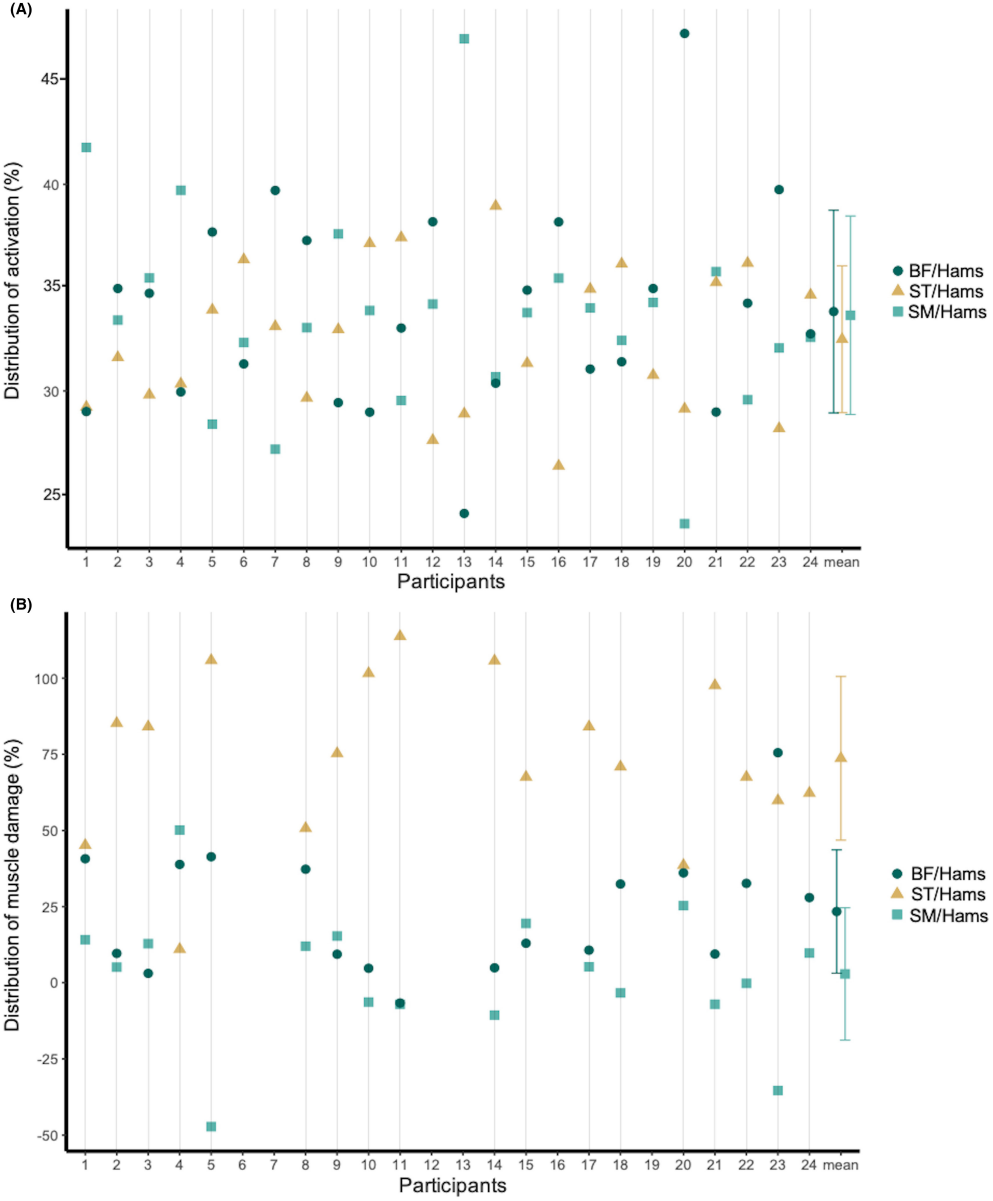
Distribution of activation (A) and distribution of damage (B) among hamstring (Hams) heads (semimembranosus, SM, square; semitendinosus, ST, triangle; biceps femoris, BF, dot). The distribution of activation (%) was calculated through the ratio of activation for each hamstring head. The distribution of damage (%) was calculated through the ratio of changes in shear modulus (before and 30 min after exercise) for each hamstring head. The distribution of damage was not calculated for participants #6, #7, #12, #13, #16, and #19 who did not exhibit a substantial change in shear modulus after the eccentric exercise [i.e., <12.6%, based on Le Sant et al^23^]

When considering normalized RMS EMG values during the eccentric exercise, we observed no substantial differences between SM (42.9% ± 9.7% RMS MAX EMG), ST (41.6% ± 9.3% RMS MAX EMG), and BF (43.1% ± 9.5% RMS MAX EMG). Accordingly, no difference between activation ratios (all *p* values = 1). Importantly, there were large interindividual differences in the distribution of activation among the hamstring heads (Figure 2A). BF/Hams was the greatest activation ratio for 10/24 participants, ST/Hams ratio was the greatest for 8/24 participants, and SM/Hams ratio was the greatest for 6/24 participants.

**FIGURE 3:**
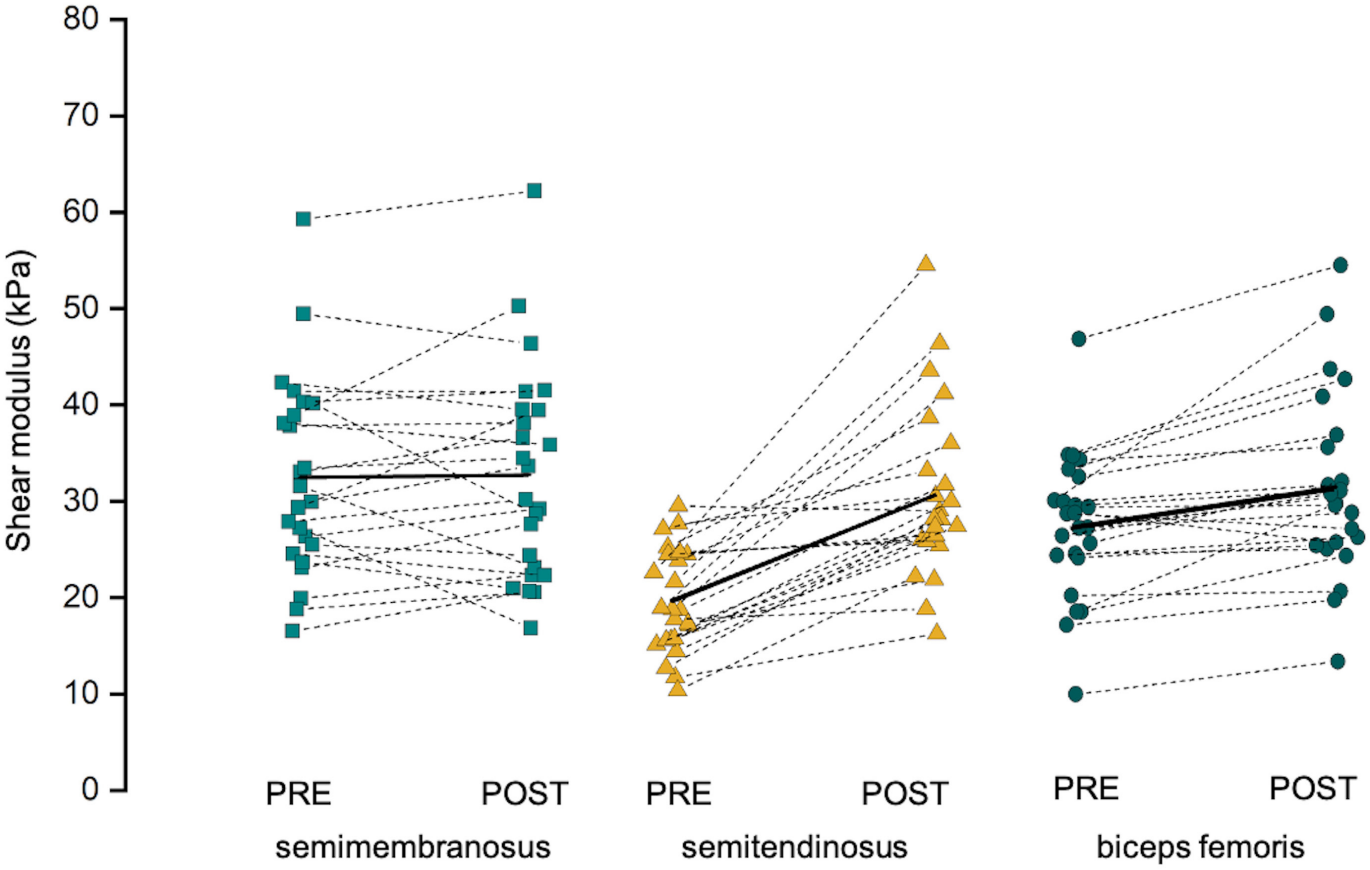
Shear modulus of the hamstring heads (semimembranosus, SM, square; semitendinosus, ST, triangle; biceps femoris, BF, dot) was measured before the eccentric exercise (PRE) and 30 min afterward (POST)

Figure 3 depicts the shear modulus values before and after eccentric exercise for each hamstring head. We found a significant muscle × time interaction for muscle shear modulus (*p* < 0.001). Bonferroni Post hoc tests revealed a significant increase in shear modulus for ST (11.0 kPa, 95% CI: 7.4–14.6 kPa, *p* < 0.001) and BF (4.1 kPa, 95% CI: 2.1–6.1 kPa, *p* < 0.001), but not for SM (0.3 kPa, 95% CI: −2.0–2.6 kPa, *p* = 1). This led to a larger distribution of damage to ST/Hams than to SM/Hams (+70.8%, 95% CI: 48.7%–92.9%, *p* < 0.001), or to BF/Hams (+50.3%, 95% CI: 29.3%–71.4%, *p* < 0.001). The distribution of damage to BF/Hams was larger than that to SM/Hams (+20.5%, 95% CI: 5.5%–35.4%, *p* = 0.046). Among the 18 participants who showed substantial muscle damage, we found that 16 had a larger distribution of damage to ST/Hams than to BF/Hams or SM/Hams (Figure 2B). It is noteworthy that the distribution of damage to ST/Hams varied between individuals (i.e., mean = 73.7%; ranged from 11.0% to 113.7%).

**FIGURE 4:**
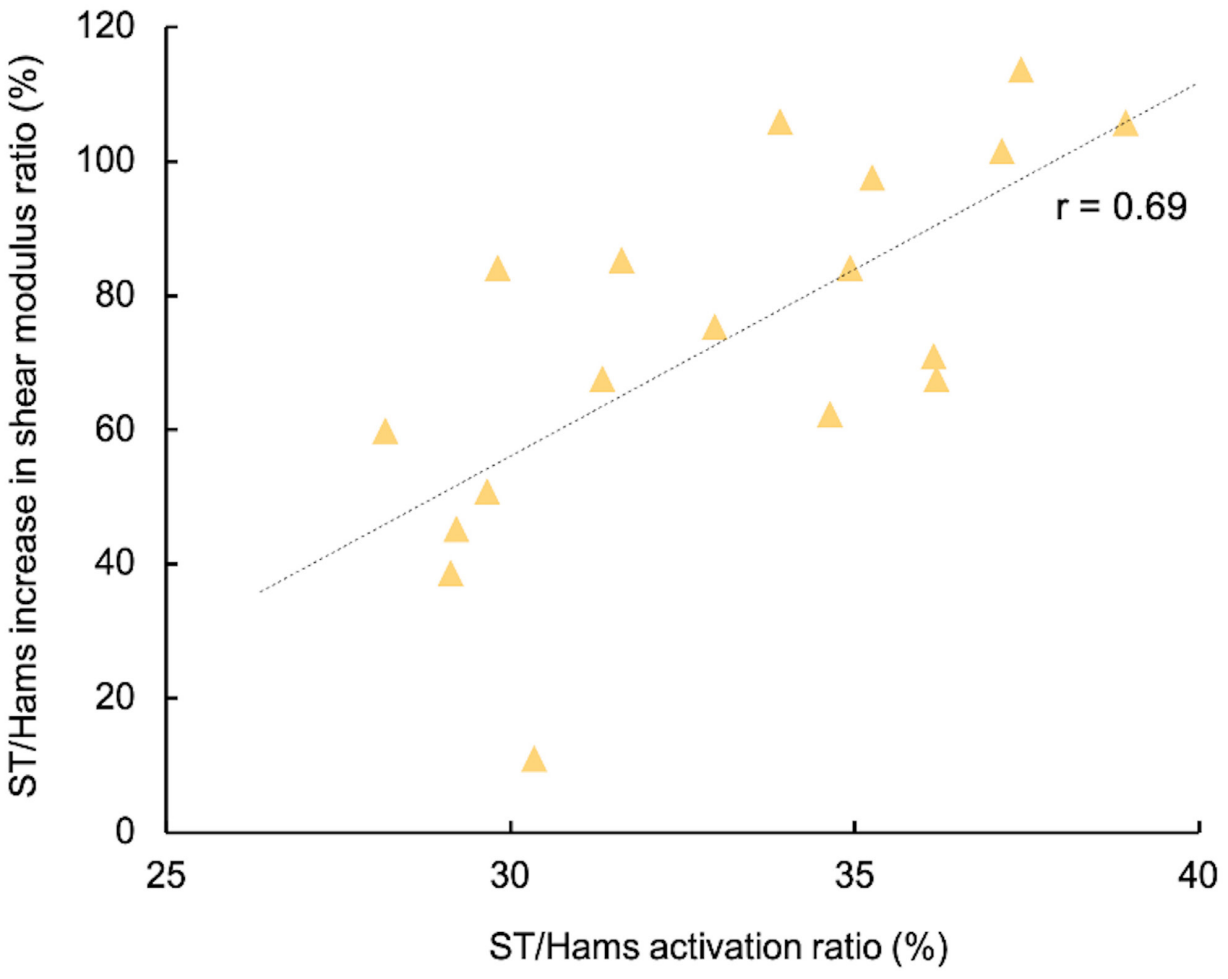
Relationship between the distribution of damage and the distribution of activation for the semitendinosus (ST/Hams). The distribution of activation (%) was calculated through the ratio of activation for ST/Hams. The distribution of damage (%) was calculated through the ratio of changes in shear modulus for ST/Hams. Hams: Hamstring

**FIGURE 5:**
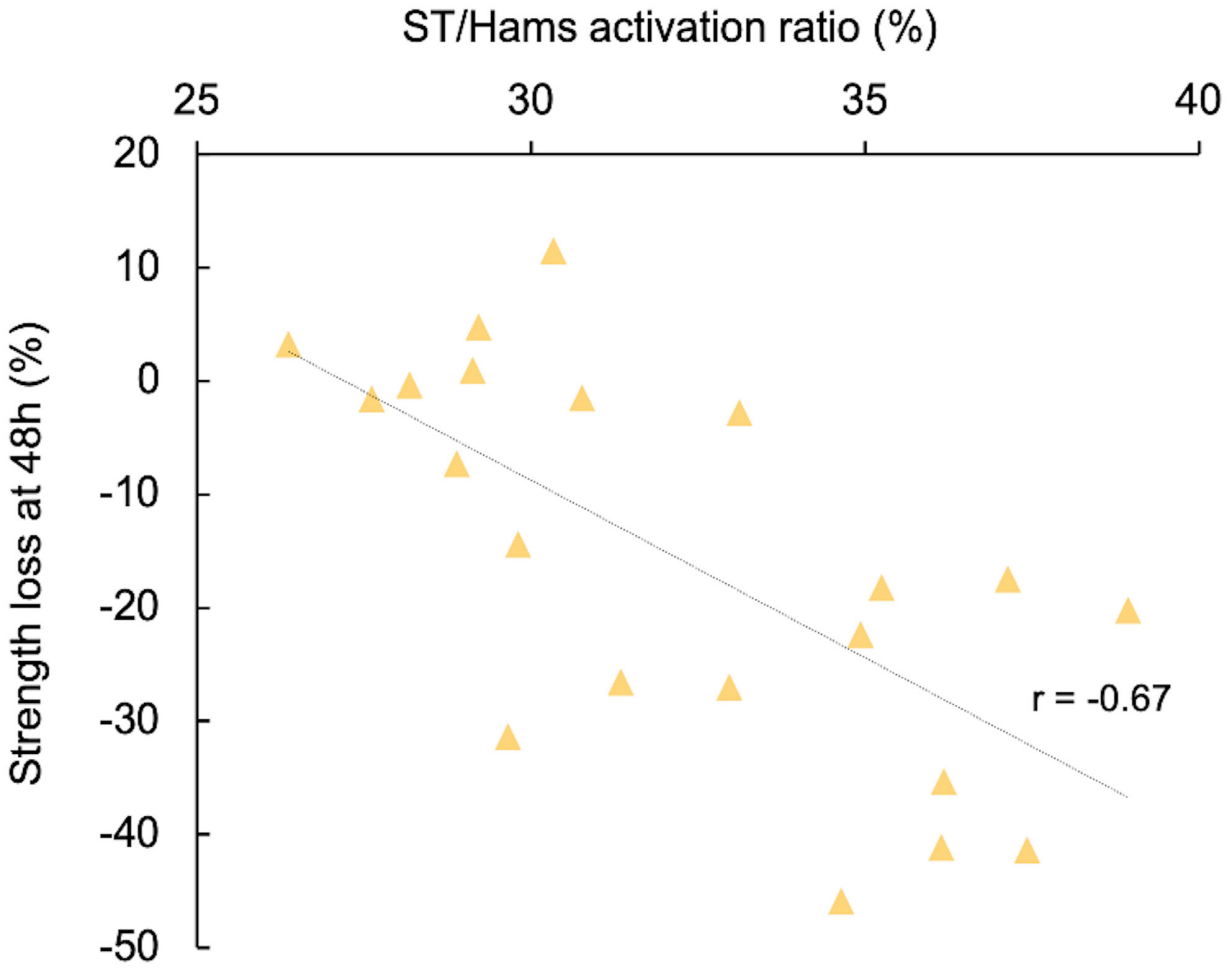
Relationship between strength loss at 48 h after eccentric exercise (%) and the ratio of activation for ST/Hams. Semitendinosus: ST; Hams: Hamstring

Pearson correlation coefficients between the distribution of activation and the distribution of damage were *large* for ST/Hams (*r* = 0.69%; 95% CI: 0.33–0.88; *p* < 0.001) (Figure 4) and *moderate* correlations for both BF/Hams (*r* = 0.45%; 95% CI: 0.034–0.76; *p* = 0.062) and SM/Hams (0.39%; 95% CI: 0.090–0.73; *p* = 0.106). This means that the greater the bias of activation is toward ST, the greater the distribution of damage to ST/Hams. Similarly, we found a *large* correlation between the distribution of the activation of the ST/Hams and the strength loss at 48 h (*r* = −0.67%; 95% CI: −0.86–−0.34; *p* < 0.001) (Figure 5), while *small* correlations were found for SM/Hams (*r* = 0.25%; 95% CI: −0.19 to 0.62; *p* = 0.26) and BF/Hams (*r* = 0.23%; 95% CI: −0.22 to 0.60 *p* = 0.31).

## 4 DISCUSSION

This study has three major findings. First, we found that in ~89% of participants there was a greater distribution of muscle damage to ST/Hams compared with BF/Hams and SM/Hams, after five sets of maximal isokinetic eccentric contractions. Second, we found that the higher the distribution of activation to ST/Hams, the larger the proportion of damage to ST/Hams (*r* = 0.69). Third, the ST/Hams activation ratio showed a *large* correlation (*r* = −0.67) with the strength loss at 48 h. This study suggests that the distribution of activation has a mechanical effect on individual muscles and that individual activation strategies should be considered as relevant information rather than noise (i.e., error of measurement).

Strength loss at 48 h after intense eccentric exercise is considered a strong indirect marker of muscle damage.^18^ This leads to the classification of the amount of muscle damage as, *mild*, *moderate*, and *severe* for <20%, 20%–50%, and >50% of strength loss at 48 h after exercise, respectively.^18^ In the current study, we found a strength loss of about 15.9% ± 16.9% at 48 h after exercise (*n* = 21), corresponding to mild damage. Accordingly, Maeo et al.^9^ reported that the one‐repetition maximal (1 RM) decreased about ~9% after 3 sets of 10 repetitions/set to 90% of 1 RM in a combination of prone and seated leg curls. In the current study, muscle damage was confirmed indirectly by the increase in the shear modulus of the hamstring heads (Figure 3). As previously demonstrated in other muscle groups,^12^, ^13^ we found a *large* relationship here between the changes in shear modulus at 30 min after exercise (averaged over the synergist muscles) and the strength loss at 48 h after exercise (*r* = −0.71). Importantly, this correlation was still *large* when considering only the changes in shear modulus of the ST (*r* = −0.70), while *moderate* and *negligible* correlations were found for BF (*r* = −0.39) and SM (*r* = −0.09), respectively. This could be explained by the largest increase in shear modulus for the ST while small and no changes were found in BF and SM, respectively (Figure 3). Overall, we confirm the strong relationship between changes in shear wave elastography and muscle strength loss following a maximal eccentric exercise.

In the current study, we found a larger increase in shear modulus in ST compared with BF and SM. This is in agreement with MRI‐based studies using T_2_ relaxation time.^7^, ^8^, ^9^, ^10^ We also found that, among the 18/24 participants that exhibited substantial muscle damage [i.e., changes in shear modulus after exercise <12.6% based on Le Sant et al.^23^ strength loss ~2.0% ± 3.3% of MVC at 48 h after exercise], 16 participants had a larger distribution of damage to ST/Hams than to BF/Hams and SM/Hams (Figure 2B). The inter‐individual variability in the ST/Hams ratio of shear modulus was noteworthy, ranging from 11%–113% (Figure 2B). This distribution of damage is meaningful, as any given strength loss can result from multiple combinations of damage distribution. A large strength loss combined with a heterogeneous distribution of damage suggests that the ability of one muscle to absorb energy during a motor task would be highly altered (Figure 2B, participant #14). This is critical when the mechanical constraints of a task impose a high amount of energy to absorb on this muscle (e.g., sprinting for hamstring muscles^30^). Although the origin of the heterogeneous distribution of damage among hamstring heads is probably multifactorial, we can speculate that the fusiform architecture of ST results in greater fiber stress compared with the pennate architecture of SM and BF muscles.^31^ It is interesting to note that the ST shows the largest increase in muscle hypertrophy after resistance training programs.^32^ Bourne et al.^32^ reported that 10 weeks of Nordic hamstring training increased the muscle volume of the BF, ST, and SM by ~6%, ~21%, and ~5%, respectively. Although muscle damage during resistance training is not required to induce muscle hypertrophy, it could be speculated that there is a link between these findings.^33^ The potential links between the distribution of damage and distribution of hypertrophy remain to be studied.

In their recent review, Bourne et al.^2^suggested that knee‐dominant exercises (e.g., Nordic hamstring and prone leg curl) favor the activation of ST, whereas hip‐dominant exercises (e.g., hip extension and stiff‐leg deadlift) favor the activation of BF and SM, based on acute T_2_ relaxation time changes after exercise. A series of studies have demonstrated that individual muscle activation strategy needs to be considered as relevant information rather than noise.^3^, ^20^, ^21^ The current study confirms that the most active muscle during maximal eccentric contraction of the knee flexors varied greatly between individuals (SM/Hams = 6 participants; ST/Hams = 8 participants; and BF/Hams = 10/24 participants). However, we found that 89% of the participants had the most damage to ST/Hams. Therefore, the mean distribution of activation only prevented us from predicting the most damaged muscle among the hamstring heads. This means that the prediction of damage localization among synergist muscles needs to consider the biomechanical features of each muscle.^34^ Nonetheless, we found that the higher the bias of activation to a muscle, the greater the distribution of shear modulus increase to this muscle (i.e., distribution of muscle damage), especially for ST/Hams (*r* = 0.69). The *moderate* correlations for both BF/Hams (*r* = 0.45) and SM/Hams (*r* = 0.39) can likely be explained by the absence/small amount of damage to this muscle. For instance, the distribution of muscle damage to SM/Hams does not exceed 50%, while some participants exhibited 100% of the damage to ST/Hams. A further is needed to determine whether this correlation exists on BF/Hams and SM/Hams when the mechanical constraints of the task favor the damage to those muscles.

Overall, this finding provides evidence that the distribution of activation has a mechanical consequence on each muscle. Also, this individual difference in the distribution of muscle activation among hamstring heads might have important functional consequences. Hence, we found a *large* correlation between the distribution of the activation to ST/Hams and the strength loss at 48 h (*r* = −0.67). Therefore, for a given overall hamstring activation, the distribution of activation to ST may lead to a greater magnitude of muscle damage. Further investigations are needed to determine whether the bias of activation to ST is decreased during the second session of eccentric to limit the functional alterations, contributing to the repeated bout effect.

There are four main limitations that require consideration. First, Gennisson et al.^35^ reported that the ability of shear wave elastography to detect changes in muscle tension decreases when the probe is placed perpendicular to the fibers. One might, therefore, think that the larger increase in shear modulus in the ST compared with BF and SM was related to muscle architecture. Indeed, ST is considered a fusiform muscle that limit the angle between fibers and ultrasound probe, while BF and SM are pennate muscles.^36^ However, MRI‐based studies using T_2_ relaxation time performed on quadriceps^37^ and hamstring^9^ muscles reported similar differences between muscles to those reported using shear wave elastography. Therefore, we are confident that the between‐muscle differences in changes in shear modulus reported in the present study are related to the differing amount of damage. Second, the relative changes in shear modulus were used to compare the amount of damage between synergist muscles. To compare individual muscles, it is recommended to calculate the index of increase in shear modulus as (i) the slope of the relationship between the changes in shear modulus before and after exercise and (ii) using the two joint angles above the slack angle.^13^ This is because the relationship between joint angle and increase in shear modulus is linear above the slack angle.^12^ In the current study, the distribution of damage between muscles was similar when considered using the relative increase in shear modulus and the index of increase in shear modulus. More precisely, we found that the index of increase in shear modulus was larger in ST (10.2%) compared to BF (2.9%, *p* = 0.035) and SM (−4.9%, *p* < 0.001). This may be because hamstring heads act at a similar relative length during knee flexion,^38^ leading to similar changes in shear modulus for a given amount of damage. For sake of clarity, we have chosen to report the relative changes in shear modulus to compare the distribution of damage among hamstring heads. Thirdly, it is well known that surface EMG is prone to crosstalk.^39^ To minimize crosstalk, we used B‐mode ultrasound to check the appropriate location of the surface electrodes, away from the border of neighboring muscles. Using this methodology, a previous showed opposite behavior of SM/Hams and ST/Hams activation ratios during Nordic hamstring exercise and stiff‐leg Deadlift^3^ which makes us confident that the crosstalk was limited. Finally, the distribution of activation among hamstring heads was calculated only during the 15 reps of the first set while a potential redistribution of activation during the following four sets was not considered. This is because EMG signals are altered by peripheral fatigue.^40^ Therefore, it is difficult to interpret the changes in EMG amplitude as a change in activation during a fatiguing task.^41^ Note that Avrillon et al.^27^ reported that the ratios of EMG of the hamstrings did not vary during a fatiguing task performed until failure. Overall, we believe that this limitation does not affect the conclusions of this study.

## 5 PERSPECTIVES

An important result is the greater distribution of damage to ST/Hams compared with BF/Hams and SM/Hams for 89% of the participants, induced by maximal eccentric contractions of the knee flexors. As this distribution was not observed for muscle activation, this underlines the large influence of the biomechanical features of each muscle on the distribution of stress among synergist muscles. This finding underlines the need to develop a non‐invasive approach to quantify the amount of stress received by each muscle to predict the distribution of muscle damage. By showing that both the magnitude of muscle damage and its distribution were correlated with ST/Hams activation ratio, we confirm that individual activation strategies should be considered as relevant information rather than noise. Further studies are needed to understand whether individual activation strategies influence the distribution of the structural adaptations within a muscle group after a training program.

## CONFLICT OF INTEREST

No potential conflict of interest was reported by the authors.

## Supporting information


Figure S1

## Data Availability

The data that support the findings of this study are available from the corresponding author upon reasonable request.
