# Supplementary figures and images for: Hamstring muscle activation strategies during eccentric contractions are related to the distribution of muscle damage
Source: Scand J Med Sci Sports. 2022 Jun 6;32(9):1335–45. doi: 10.1111/sms.14191 (PMC9541962; doi:10.1111/sms.14191)

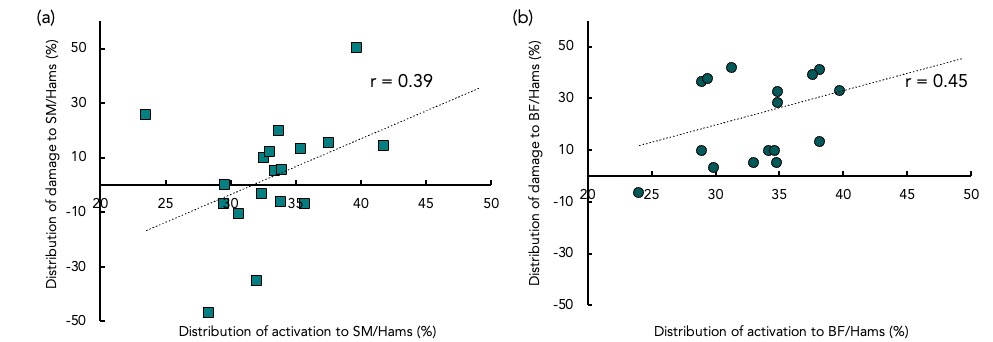

Supplement: Supplementary file 1 — Figure S1 [file SMS-32-1335-s001.jpg]
